# Loxoscelism leading to penile necrosis

**DOI:** 10.1590/0037-8682-0516-2021

**Published:** 2021-12-17

**Authors:** Gabriel Chahade Sibanto Simões, Joao Roberto Paladino, Alexandre Gomes Sibanto Simões

**Affiliations:** 1 Universidade Estadual de Campinas, Departamento de Cirurgia, Programa de Residência Médica em Área Básica Cirúrgica, Campinas, SP, Brasil.; 2 Fundação ABC, Faculdade de Medicina, Departamento de Urologia, Santo André, SP, Brasil.

**FIGURE 1:**
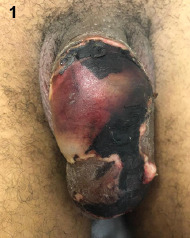
Circumferential penile lesion consisting of erythema, ischemia, and necrosis, associated with significant loss of tissue.

Brown spider (genus Loxosceles) bites are frequent in South America, occurring in the upper limbs, thorax, and inner thigh. Genital area involvement is rare, with few reports in the literature^1^. A 23-year-old patient sought medical care 12 hours after being bitten by a brown spider. The patient experienced an exacerbation of erythema associated with pain and was advised to apply cold compresses to the wound and take a non-steroidal anti-inflammatory drug and cephalexin antibiotic for seven days. A week later, the patient returned complaining of increased pain. The penile lesion measured approximately 7 cm and was surrounded by erythema, ischemia, and necrosis (Figure 1). The patient had no systemic symptoms. 

**FIGURE 2:**
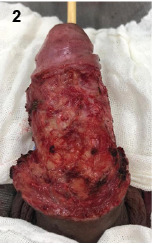
Patient’s penis after surgical debridement and removal of all devitalized tissue, consisting of granulation tissue in good appearance.

**FIGURE 3:**
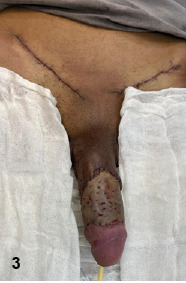
The immediate postoperative result after a total mesh skin graft is performed, using the left and right iliac donor areas.

Surgical debridement was performed (Figure 2). The patient underwent broad-spectrum antibiotic therapy. After 14 days, granulation of the tissue is noted; however, extensive loss of penile body tissue required a total mesh skin graft reconstruction using the left and right iliac donor areas^2^ (Figure 3).

Loxosceles bite is characterized by two painless punctiform orifices, progressing to an erythematous plaque with central clearing. After minutes or hours, a local reaction with intense pain accompanied by erythema, edema, vesicles, and blisters may occur. However, severe and systemic symptoms are uncommon; necrotic lesions are rare, measuring 1-2 cm, and typically resolve within weeks. Surgical debridement and reconstruction are rarely necessary^3^. In this case, the lesion was associated with extensive and deep necrosis of the penile shaft. Surgical debridement was necessary to avoid infectious complications, loss of function, and to shorten the recovery time.
